# Proteomic Study Revealed a Distinction Between Human Dermal Fibroblasts and Mesenchymal Stem Cells from Different Sources

**DOI:** 10.1007/s12015-025-10926-4

**Published:** 2025-06-27

**Authors:** Slavomíra Nováková, Zuzana Hatoková, Maksym Danchenko, Gábor Beke, Ľuboš Kľučár, Lucia Slovinská, Denisa Harvanová, Peter Baráth, Ján Strnádel, Erika Halašová, Henrieta Škovierová

**Affiliations:** 1 Biomedical Centre Martin, Jessenius Faculty of Medicine in Martin, Comenius University in Bratislava, Malá Hora 4C, Martin, 036 01 Slovakia; 2 Plant Science and Biodiversity Center, Slovak Academy of Sciences, Dúbravská cesta 9, Bratislava, 845 23 Slovakia; 3 Institute of Molecular Biology, Slovak Academy of Sciences, Dúbravská cesta 21, Bratislava, 845 51 Slovakia; 4 Associated Tissue Bank, Faculty of Medicine, Pavol Jozef Safarik University and Louis Pasteur University Hospital, Trieda SNP 1, Košice, 04011 Slovakia; 5 Institute of Chemistry, Slovak Academy of Sciences, Dúbravská cesta 9, Bratislava, 845 38 Slovakia

**Keywords:** Cell-based therapy, HDFa, Proteome profiling, Surface markers, Tissue regeneration

## Abstract

Mesenchymal stem cells (MSCs) are an essential tool in cell-based therapies. One of the most crucial factors for efficacy in regenerative medicine is the source of MSCs. Tissue origin has long been suggested as a potential determinant of MSC properties. Human dermal fibroblasts (HDFa) share similar characteristics with MSCs, and the question of whether HDFa are functionally equivalent to MSCs remains debated. The present work used proteomic and phenotypic analyses to compare HDFa, dental pulp stem cells (DPSCs), and adipose-derived mesenchymal stem cells (AD-MSCs). We observed similarities and/or differences in morphology, cell surface markers, differentiation, and proteomic profile. Proteome was profiled by nano liquid chromatography and comprehensively quantified by mass spectrometry. In fact, HDFa and MSCs shared similar surface markers, growth kinetics, and differentiation capacity. Proteomic analysis reproducibly identified and quantified 3,051 proteins, 86 of them were differentially abundant according to strict statistical criteria. We identified a set of proteins that determined signatures for each stem cell origin. Gene Ontology (GO) term enrichment of differentially accumulated proteins, and Gene Set Enrichment Analysis (GSEA) identified signaling pathways characteristic to individual cell types. Particularly, we highlighted signaling pathways involved in cell migration, adhesion, and Wnt signaling as downregulated in HDFa compared to DPSCs. Angiogenesis and vascularization were explicitly associated with AD-MSCs. The tissue repair process requires a well-coordinated integration of complex molecular events, including cell migration and proliferation, extracellular matrix deposition, angiogenesis, and remodeling. We propose that HDFa are an alternative to MSCs, but predict their worse behavior in defect repair models compared to DPSCs. Plausibly, AD-MSCs are more suitable candidates for angiogenesis models compared to DPSCs.

**Graphical Abstract:**

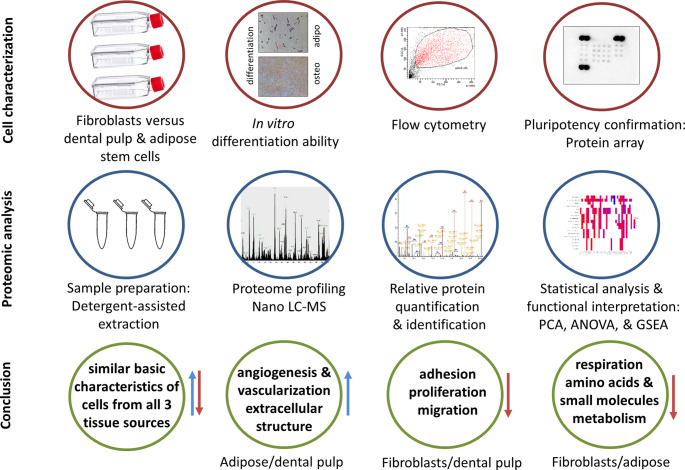

**Supplementary Information:**

The online version contains supplementary material available at 10.1007/s12015-025-10926-4.

## Introduction

Mesenchymal stem cells (MSCs) are a proven tool in regenerative medicine, which aims to restore damaged tissues and thus offer new therapeutic options for treating several diseases and injuries. Since their introduction in regenerative medicine, preclinical and early clinical studies have steadily increased over the past decade [1, 2]. In fact, MSCs are self-renewing multipotent stromal cells derived from early mesoderm that can differentiate into various cell types, including osteocytes, chondrocytes, and adipocytes. Initially, they were discovered by Friedenstein and isolated from human bone marrow (hBM-MSCs) [3]. Since then, MSCs have been found and isolated from various adult and perinatal tissues, making such diversity one of their unique characteristics [4, 5].

The clinical application of MSCs is the subject of intense debate. The therapeutic potential of MSCs is primarily associated with their ability to migrate to sites of injury (homing), adhere, and integrate (engrafting) into damaged tissues. Subsequently, MSCs can directly differentiate into specialized cell types or act via immune modulation by secreting protective growth factors, cytokines, and extracellular vesicles. They modulate the local microenvironment and promote healing, even without direct differentiation into target tissues [6–8]. The biological mechanisms of MSCs action have not yet been fully elucidated. A significant advantage of MSCs is their low immunogenicity, enabling autologous transplantation, reducing the risk of rejection, and enhancing therapeutic efficacy [9, 10]. It is precisely because of the reduced need for immunosuppression in recipients that MSCs are particularly valued in cell-based therapies.

Despite the properties mentioned above, using MSCs in regenerative medicine is not without problems. The heterogeneity of MSCs can lead to variability in their therapeutic efficacy. Differences in donor health status, age, and source tissue can affect the quality and function of MSCs, complicating their clinical use [5]. For safe and effective cell therapy, it is important to ensure sufficient proliferation, differentiation, and migration capacity, as well as cellular uniformity. One of the most crucial factors for efficacy in regenerative medicine is the source of MSCs. Some sources of MSCs are more suitable for particular organ regeneration than others [11]. Isolation from bone marrow is relatively invasive; therefore, new tissues of MSC origin appeared, such as dental pulp, adipose tissue, umbilical cord tissue, Wharton’s jelly, amniotic fluid, and others [12].

One potential alternative source of MSCs is human dermal fibroblasts adult (HDFa). They are adherent cells with an elongated spindle-shaped morphology vital for tissue development, maintenance, and repair. With such properties, they meet the characteristics of MSCs, according to International Society for Cellular Therapy (ISCT) [13]. Criteria were updated later: the committee reaffirmed the importance of standardized cell nomenclature and emphasized the need for additional reporting criteria, such as tissue of origin and functional assays, to more accurately characterize MSCs [14]. No single marker is explicitly specific for MSCs [15]. Some authors hypothesized that HDFa are essentially senescent MSCs of the same cell type [16, 17]. Several studies have shown that MSCs from different sources, including HDFa, have only minimal differences. Although indistinguishable with conventional markers, they could significantly differ in immunomodulation, proliferative and differentiation capacity, and specific flow cytometry markers [5, 18, 19].

We aspired to reveal differences and similarities between HDFa, dental pulp stem cells (DPCSs), and subcutaneous adipose-derived mesenchymal stem cells (AD-MSCs) cultivated in vitro under the same conditions. We demonstrated how stem cell origin influenced proteome profiles. Next, we highlighted a signature proteomic pattern for HDFa and two other types of MSCs. Finally, we focused on identifying CD markers for flow cytometry that would distinguish individual types of cell origin and could be implemented as potential stratifying features to authenticate the identity of the isolated cells.

## Materials and Methods

### Culture of hMSCs from Different Sources

hMSCs (hDPSCs and hAD-MSCs) and HDFa were obtained from 3 independent donors. HDFa were purchased from Gibco (NY, USA), and DPSCs from Lonza (Switzerland) as cryopreserved cells, while AD-MSCs isolated from subcutaneous aspirate were kindly provided by the Tissue Bank of Pavel Jozef Safarik University in Košice, Slovakia. Then, cells were cultured in a humidified incubator at 37 °C and 5% CO_2_ in DMEM-F12 cultivation medium (Gibco, NY, USA) supplemented with 10% FBS (Biosera, France), 100 U/mL penicillin, and 100 µg/mL streptomycin (Biosera, France). During preculture, after reaching 80–90% confluence, cells were harvested using TrypLE Express Enzyme (Thermo Fisher Scientific, MA, USA) and then seeded at 5 × 10^3^ cells/cm^2^. Cell morphology was monitored during all experiments using the phase contrast microscope XDS-2 (Optika, Italy). All analyses were performed with passage 5.

### Cell Viability Assay

Cells of each type were seeded in 96-well plates (Corning, NY, USA) at 5 × 10^3^ cells/cm^2^ density in hexaplets. At days 2, 4, and 6 the cells were washed with Dulbecco’s PBS solution (DPBS, Gibco, NY, USA) and incubated with a mixture of 3-(4,5-dimethylthiazol-2-yl)−2,5-diphenyltetrazolium bromide solution (MTT, Duchefa Biochemie, Netherlands) and culture medium in ratio 1:10 for 5 h at 37 °C in a humidified atmosphere. Then, 10% SDS solution (Sigma-Aldrich, MO, USA) was applied to each well, and the plates were incubated overnight. Changes were measured spectrophotometrically at 570 nm using a microplate reader (BioTek, VT, USA).

### Stemness Evaluation of MSCs

#### In Vitro Multilineage Differentiation

Control and differentiated cells were grown in 6-well plates. On the 21st day after osteogenic (OsteoMAX-XF differentiation medium, Sigma-Aldrich, MO, USA) or adipogenic (StemPro adipogenesis differentiation kit, Gibco, NY, USA) medium application, cells were fixed by 4% paraformaldehyde (Cell Signaling Technology, MA, USA) for 30 min at room temperature (RT). After DPBS washing, formed Ca^2+^ deposits or lipid-rich vacuoles were stained with Alizarin Red S solution (Merck, Germany) for 3 min or with Oil Red O solution (Sigma-Aldrich, MO, USA) for 30 min at RT, respectively. Next, the dye was aspirated, and cells were carefully washed 3 times with DPBS or ultrapure water to remove the remaining stain. Finally, cells were kept in ultrapure water. Staining intensity was monitored and micrographs were captured by the inverted microscope XDS-2 (Optika, Italy).

#### Cell Surface Marker Analysis By Flow Cytometry

When cells reached 80% confluence, a single-cell suspension was prepared by passing the cells through a 70 μm strainer (Corning, NY, USA). After centrifugation (400 × *g*, 13 °C, 5 min), 1 × 10^5^ cells were incubated in FACS buffer (1 mM EDTA and 5% mouse serum in DPBS) for 30 min at 4 °C in the dark. After incubation, 5 µL of a fluorescently labeled antibody (BioLegend, CA, USA) was added to 1 × 10^5^ cells, followed by another incubation for 1 h at 4 °C in the dark. Then, the labeled samples were washed with DPBS buffer and centrifuged at 450 x *g*, 13 °C, 4 min. Stained cell pellet was resuspended in FACS buffer. All samples were analyzed using the FACS Aria II cytometer (BD Biosciences, NJ, USA) on 10,000 events and BD FACSDiva software v8.0.1. Gating was set up based on an unstained/isotype control, and the gating of all samples was consistent.

#### Pluripotent Proteins Detection

Abundances of pluripotent proteins were determined by Proteome Profiler Human Pluripotent Stem Cell Array (R&D system, MN, USA) according to the manufacturer’s instructions. This array provides a targeted analysis of 15 proteins, established markers of pluripotency. Briefly, cultured MSCs were washed with DPBS and scraped off into lysis buffer 16 supplemented in the kit. Total protein concentration was detected using micro BCA protein assay kit (Thermo Fisher Scientific, MA, USA). The same amount of total protein (270 µg) from different cell types was used for parallel incubation. Proteins were detected by chemiluminescence after application of the developing solution (ClarityMax, Bio-Rad, CA, USA) and visualized using ChemiDoc XRS + system (Bio-Rad, CA, USA) as described previously [20]. Densitometric analysis of protein abundances was accomplished via ImageLab software v6.0 (Bio-Rad, CA, USA). Signal intensities (pixel densities) were normalized to the positive control.

### Proteomic Analysis

#### Protein Extraction and Digestion

Cells were harvested in biological triplicates on a day when cells reached 80‒90% confluence. Then, cells were washed with ice-cold DPBS and scraped off from flasks using SDT buffer (4% SDS, 0.1 M Tris pH 7.6, and 100 mM dithiothreitol) supplemented with protease (Roche, Switzerland) and phosphatase (Sigma-Aldrich, MO, USA) inhibitors. Then, lysates were transferred to the tubes, incubated on ice for 15 min, and sonicated (3 × 5 s, 20% pulse), followed by 20 min centrifugation (14,000 × *g*) at 13 °C. The final supernatant was aliquoted and stored at − 80 °C until use. Protein concentration was determined by Pierce 660 nm kit (Thermo Fisher Scientific, MA, USA).

Protein digestion was performed on beads as described in the literature [21]. Aliquots of 60 µg of protein samples were alkylated with 20 mM iodoacetamide in the dark for 30 min at RT and 600 rpm on thermoshaker TS-100 C (Biosan, Latvia) and quenched with 5 mM dithiothreitol. Next, the mixture (1:1) of beads was prepared: Sera-Mag Carboxylate-Modified Beads (Cytiva, UK), hydrophilic solids 50 µg/µL, and hydrophobic solids 50 µg/µL. On the magnetic rack MagneSphere (Promega, WI, USA), the beads were pelleted for 1 min, and the supernatant was removed by pipette. Off the rack, the beads were reconstituted in bidistilled water and pipette-mixed. Following, 30 µL (60 µg) of the bead stock was added to the alkylated/quenched protein samples and pipette-mixed. For binding, 70 µL of 100% ethanol was added to the suspension to achieve 50% final ethanol concentration. The tubes were incubated in the thermoshaker for 10 min at RT and 1000 rpm. The beads were pelleted for 2 min, and the supernatant was discarded. Bond proteins were washed 5 times with 140 µL of 80% ethanol. Next, 10 µL of 0.1 µg/µL trypsin (Promega, WI, USA) and 50 µL of 50 mM ammonium bicarbonate were added. The samples were incubated in the thermoshaker for 18 h at 37 °C and 1000 rpm. Consequently, the beads were pelleted for 2 min, and the supernatant was recovered into a fresh 2 mL tube, followed by a second elution with 60 µL of 50 mM ammonium bicarbonate. The peptide concentration was measured with a microvolume spectrophotometer DS-11 FX+ (Denovix, DE, USA).

#### Relative Label-Free Quantification By Liquid Chromatography Coupled Mass Spectrometry

Chromatographic separation was done on Vanquish Neo (Dionex Softron for Thermo Scientific, Germany) at 35 °C and flow rate 250 nL/min. Peptides were desalted on PepMap Neo trap C18, 300 μm × 5 mm, 5-µm particle size (Thermo Fisher Scientific, Lithuania) at 50 µL/min and profiled on EASY-Spray PepMap Neo C18, 75 μm × 500 mm, 2-µm particle size (Thermo Fisher Scientific, Lithuania) in 2-step gradient 2–24% in 100 min and 24–40% in 20 min of mobile phase (80% acetonitrile and 0.1% formic acid). Mass spectra were collected on Orbitrap Exploris 240 (Thermo Fisher Scientific, Germany) in data-dependent mode with 2 s cycle at top speed and 2 kV on capillary with internal mass calibration at run start. Parent scan 350–1,700 m/z was done at 120,000 resolution with an automatic gain control (AGC) target 200% and a maximum injection time 50 ms. The precursor intensity threshold was set to 5 × 10^4^ with 60 s exclusion and 30% high-energy collisional dissociation (HCD) fragmentation. Fragments were acquired at a resolution 30,000, an AGC target 300%, and a maximum injection time 200 ms.

The generated dataset was processed in MaxQuant v1.6.17.0 [22] with Andromeda search engine. Carbamidomethylation C was set as a permanent modification, and oxidation M plus deamidation N/Q were selected as variable modifications. Peptide tolerance in the first search was 10 ppm, and in the main search after recalibration was 2 ppm, while the fragment tolerance was 10 ppm. Based on the reverse decoy database search, the false discovery rate for peptides and proteins was 1%. Match between the runs and label-free quantification (LFQ) were activated. Human proteome sequences (104,573 entries) downloaded from UniProt (uniprot.org) in May 2024 were used for the database search.

### Western Blot

Protein aliquots (35 µg) from each sample, prepared in Laemmli buffer (Bio-Rad, CA, USA), were loaded onto Tris-glycine gradient gels (4–20%) and separated using a Mini-PROTEAN Tetra Cell chamber (Bio-Rad, CA, USA). Electrophoresis was conducted at 60 V for 20 min, followed by 200 V until the tracking dye reached the bottom of the gel. Subsequently, the separated proteins were transferred onto a 0.45 μm nitrocellulose membrane (Thermo Fisher Scientific, MA, USA) using a semi-dry blotting unit (Biometra, Germany) under a transfer condition of 1 mA/cm². The membrane was blocked (5% BSA in Tris-buffered saline with Tween 20, TBS-T) for 1 h at RT and incubated overnight with primary antibodies to target or reference proteins at 4 °C. Antibodies were diluted in 5% non-fat dry milk. After incubation, the membrane was thoroughly washed with TBS-T 3 times and further incubated with horseradish peroxidase-conjugated goat anti-rabbit IgG (#7074, Cell Signaling Technology, MA, USA, dilution 1:2,000) or horseradish peroxidase-conjugated goat anti-mouse IgG (#ab6789; Abcam, UK, 1:2,000) for 2 h at RT. Proteins were detected by chemiluminescence after application of the developing solution according to manufacturer’s instructions (Thermo Fisher Scientific, MA, USA) and visualized using ChemiDoc XRS + system (Bio-Rad, CA, USA). Densitometric analysis of protein abundance was accomplished via ImageLab software v6.0 (Bio-Rad, CA, USA). Signals were normalized to glyceraldehyde 3-phosphate dehydrogenase (GAPDH, Santa Cruz, TX, USA, sc-166574, dilution 1:750). The following antibodies were used: CD13 (Cell Signaling Technologies, MA, USA, #32720, dilution 1:750) and CD9 (Cell Signaling Technologies, MA, USA, #13403, dilution 1:500).

### Data Analysis and Bioinformatics

Statistical analysis of proteomic data was done in Perseus v1.6.15 [23] using ANOVA corrected for multiple testing by permutation followed by Tukey’s posthoc test. The output protein Group table from MaxQuant was filtered for reverse proteins, contaminants, and low-confidence proteins. After log_2_ transformation of the LFQ intensities, only proteins with 3 valid values in at least 1 experimental group were retained, and missing values were imputed from a lower range of the normal distribution curve. The principal component analysis (PCA) was used to evaluate sources of variability among samples and replicates.

To determine the statistical significance of cell proliferation parameters, surface markers, pluripotent proteins, and from densitometric evaluation of proteins determined by western blot, we used the Kruskal-Wallis followed by Dunn’s post-hoc test. The significance criterion was a corrected *p* ≤ 0.05 (Bonferroni correction). Statistical analysis was evaluated using Statistic Kingdoms software (https://www.statskingdom.com/140MeanT2eq.html).

Next, Gene Ontology (GO) analyses were conducted using the R programming language [24] and the clusterProfiler package [25–28]. Gene lists, derived from pairwise comparisons of proteomics data, were input into the enrichGO function. This function employs a hypergeometric test to assess whether specific GO terms are significantly over-represented in the target gene list compared to a defined background universe. Analyses were performed separately for the three GO categories—biological process (BP), molecular function (MF), and cellular component (CC)—using human gene annotations from the org.Hs.eg.db package [29]. Also, Gene Set Enrichment Analysis (GSEA) for the three GO categories (BP, MF, CC) was performed using the gseGO function from the clusterProfiler package, which leverages a ranked gene list based on log_2_ fold-change values to assess the enrichment of GO terms. The resulting enrichment data were visualized with the enrichplot package using ridge plots, tree plots, and heatmaps [25–28].

## Results

### hMSCs from Different Tissue Sources and HDFa Exhibited Similar Characteristics

**Fig. 1:**
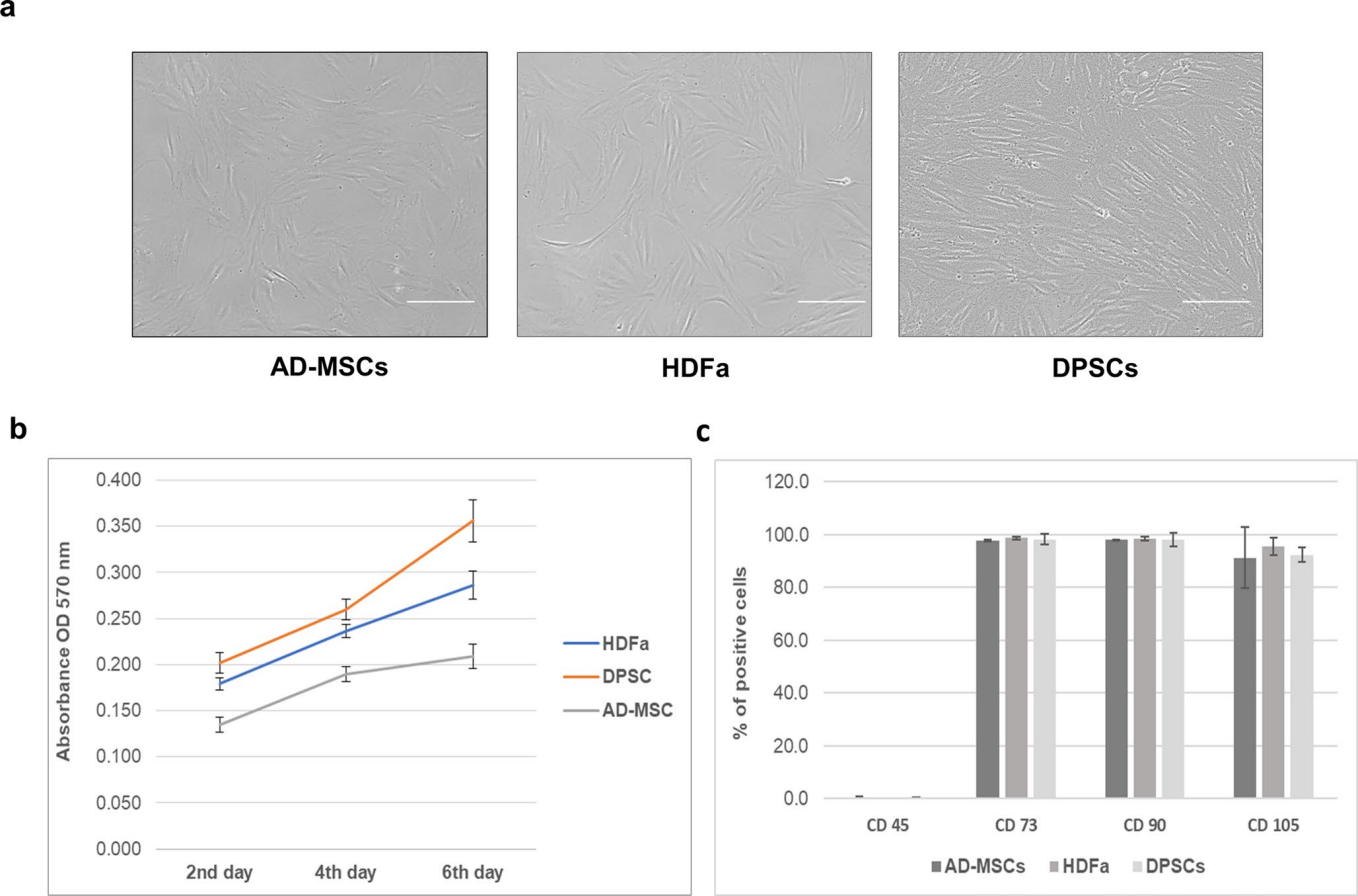
Characterization of HDFa, DPSCs, and AD-MSCs. **a** Representative micrographs taken by phase-contrast microscopy showing the morphologies of cells. Scale bar is 100 μm. **b** Growth curve analysis between day 2 and day 6 using MTT proliferation assay. No significant differences were observed on any day. **c** Flow cytometry analysis of essential CD73, CD90, and CD105 markers contrasting negative marker CD45 showed no significant differences. Data is presented as mean ± SD, *n* = 3 per tissue source

Before applying comparative untargeted proteomic analysis, we focused on confirming the fundamental characteristics of MSCs. Human MSCs from 2 different tissue sources (DPSCs and subcutaneous AD-MSCs) and HDFa were cultivated in the same conditions using DMEM-F12 medium. Then, they were validated with reference to ISCT guidelines. Cells were adhered to plastic culture plates and exhibited fibroblast-like shape morphology (Fig. 1a, Fig. S1). The critical parameter of MSCs quality is the ability to grow and proliferate in vitro. A similar pattern of growth curve was observed for cells from all 3 sources during the first 6 days; in fact, we spotted no significant difference in proliferation dynamics (Fig. 1b). Even on day 6, when cell expansion showed the greatest distances from each other compared to previous days, the comparison of absorbances showed no significant changes. Cells were harvested for further analysis when they reached ~ 80% confluence. Flow cytometry analyses identified all sources as positive for essential surface mesenchymal stem cell markers CD73, CD90, and CD105 as well as negative for CD45 (Fig. 1c).

**Fig. 2:**
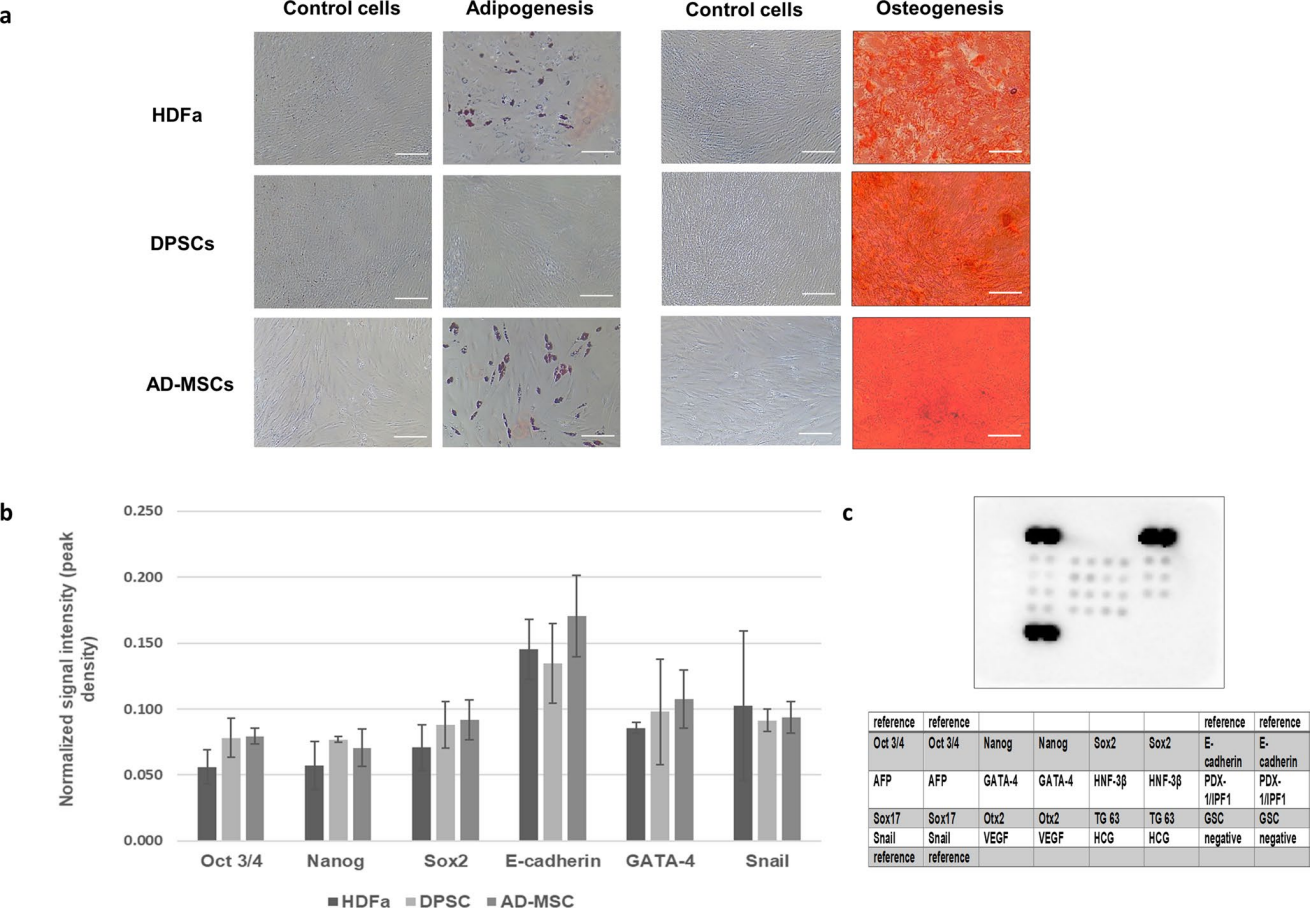
Stemness evaluation of HDFa, DPSCs, and AD-MSCs. **a** Representative images of in vitro differentiation ability on the 21st day of cultivation. Scale bar is 100 μm. **b** Quantification of pluripotency markers via antibody array. Data showed no significant differences. Data are presented as means ± SD, *n* = 3 per tissue source. **c** Representative antibody array

The percentage of positive markers was more than 90%, with a negative marker present in less than 1%. HDFa and MSCs revealed the potential to differentiate into a mesodermal lineage. Calcium deposits and lipid-rich vacuoles accumulated within the cells by day 21 following the application of the differentiation medium, indicating osteogenesis and adipogenesis, respectively (Fig. 2a). Only DPSCs showed no adipogenic differentiation, corresponding to the findings of another research group [30, 31].

Then, the pluripotency of MSCs was confirmed by targeted detection of markers using Proteome Profiler. From the 15 proteins included in the assay, we focused on key proteins of pluripotency such as Oct3/4, Sox2, Nanog, GATA-4, Snail, and higher expressed E-cadherin (Fig. 2b and c; Fig. S2). Since the concentration of these proteins in the samples is very low (up to 270 µg of protein was required), untargeted proteomics did not detect any of the 15 listed proteins. Our results showed no significant differences in protein abundances between cellular sources. Using the same array, another group [32] observed significantly higher abundances of E-cadherin, PDX, Sox2, and GSC only in DPSCs; other MSCs showed similar results to our case. Overall, the results described in this section indicated that HDFa, DPSCs, and AD-MSCs are very similar in fundamental characteristics.

### Deep Untargeted Proteomic Analysis of MSCs

**Fig. 3:**
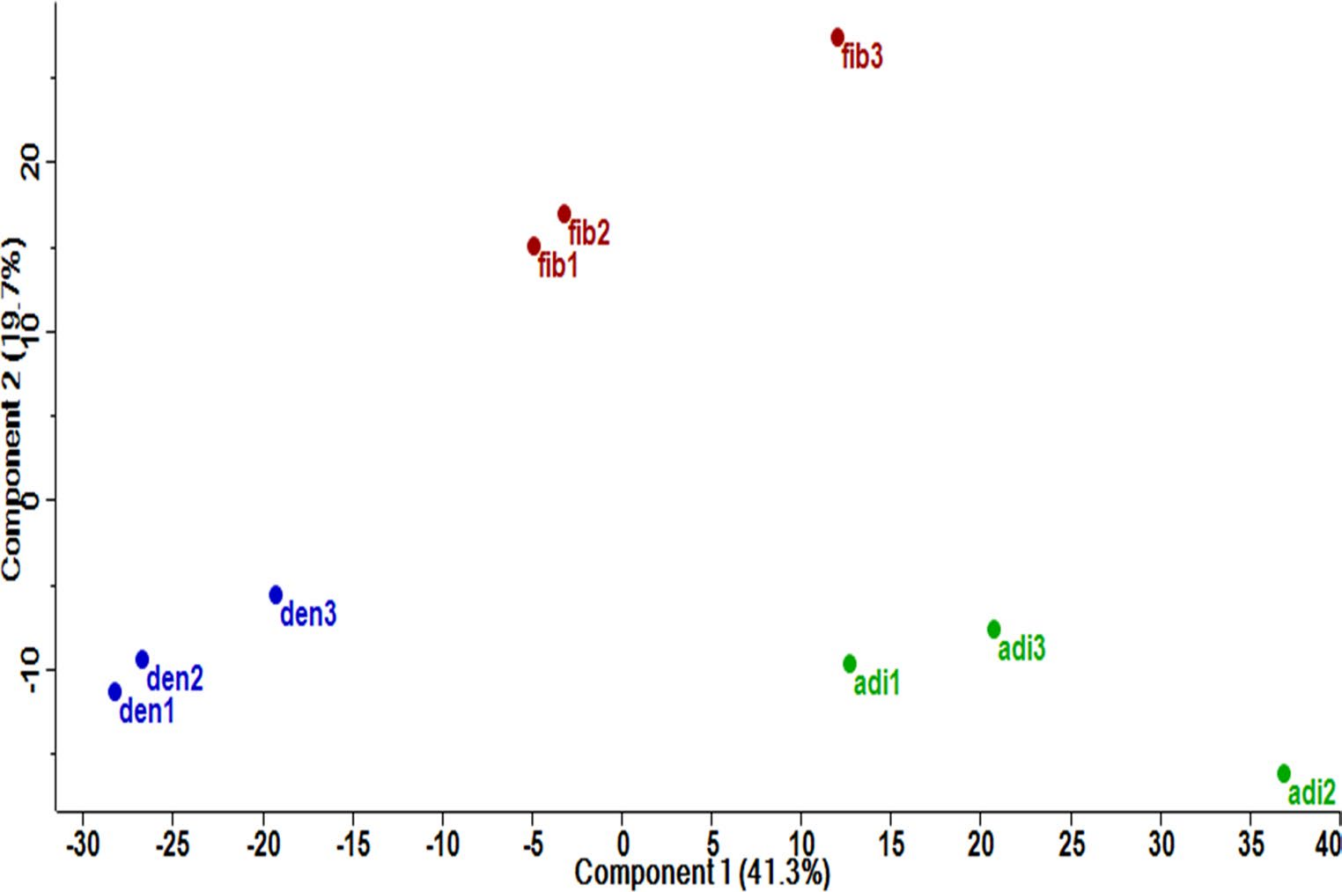
Principal component analysis of all quantified proteins for each MSC source (*n* = 3 per tissue source): fib (HDFa), adi (AD-MSCs), and den (DPSCs)

When characteristics of the cell types were established, we proceeded to the comprehensive untargeted analysis of cellular proteomes using nano liquid chromatography coupled mass spectrometry and data processing in MaxQuant-Perseus, enabling robust and accurate relative quantification and associated statistical analysis. Overall, we identified 4,347 proteins and quantified 3,051 (Table S1). We applied strict statistical filtering ANOVA *Q* ≤ 0.01 followed by Tukey’s *P* ≤ 0.01 and less than 35% coefficient of variation complemented by effect size threshold log_2_ ratio ≥ 1. As a result, we revealed 86 differentially accumulated proteins in stem cells of various origins. The pattern of protein abundances showed relevant similarities between HDFa, DPSCs, and AD-MSCs. Principal component analysis of all quantified proteins revealed clustering of biological replicates in two-dimensional space, confirming excellent reproducibility. It indicated that the difference between the proteome of HDFa and other MSCs explained ∼ 20% of data variance in component 2, while component 1 separated AD-MSCs from DPSCs, explaining ∼ 41% of data variance (Fig. 3). All 3 stem cell sources were clearly separated, while intrasource variability was much higher than intradonor variability. Differentially abundant proteins were bioinformatically analyzed upon stringent filtering to reveal plausible functional implications.

### Functional Analysis of Differentially Regulated Proteins in HDFa and Adult MSCs by GO Enrichment Analysis

Comparing HDFa and DPSCs, we revealed 23 accumulated and 18 depleted proteins. In contrast, 12 proteins accumulated and 12 proteins depleted in HDFa versus AD-MSCs. Notably, 33 proteins were more abundant, and 34 were less abundant in AD-MSCs than DPSCs. These findings indicate that despite considerable similarities in proteomes, there are also differences in the amount of specific proteins (Table S1). We argue that these proteins might explain key unique features of individual cell lineages. Next, GO enrichment analyses of biological processes were performed on the differentially abundant proteins from each comparison to determine dominant affected functions. We did not find any specific substantial enrichment for HDFa versus AD-MSCs comparison. We observed a very similar pattern, which likely reflects the fact that these two cell types evolved from a common fibroblast progenitor [33].

**Fig. 4:**
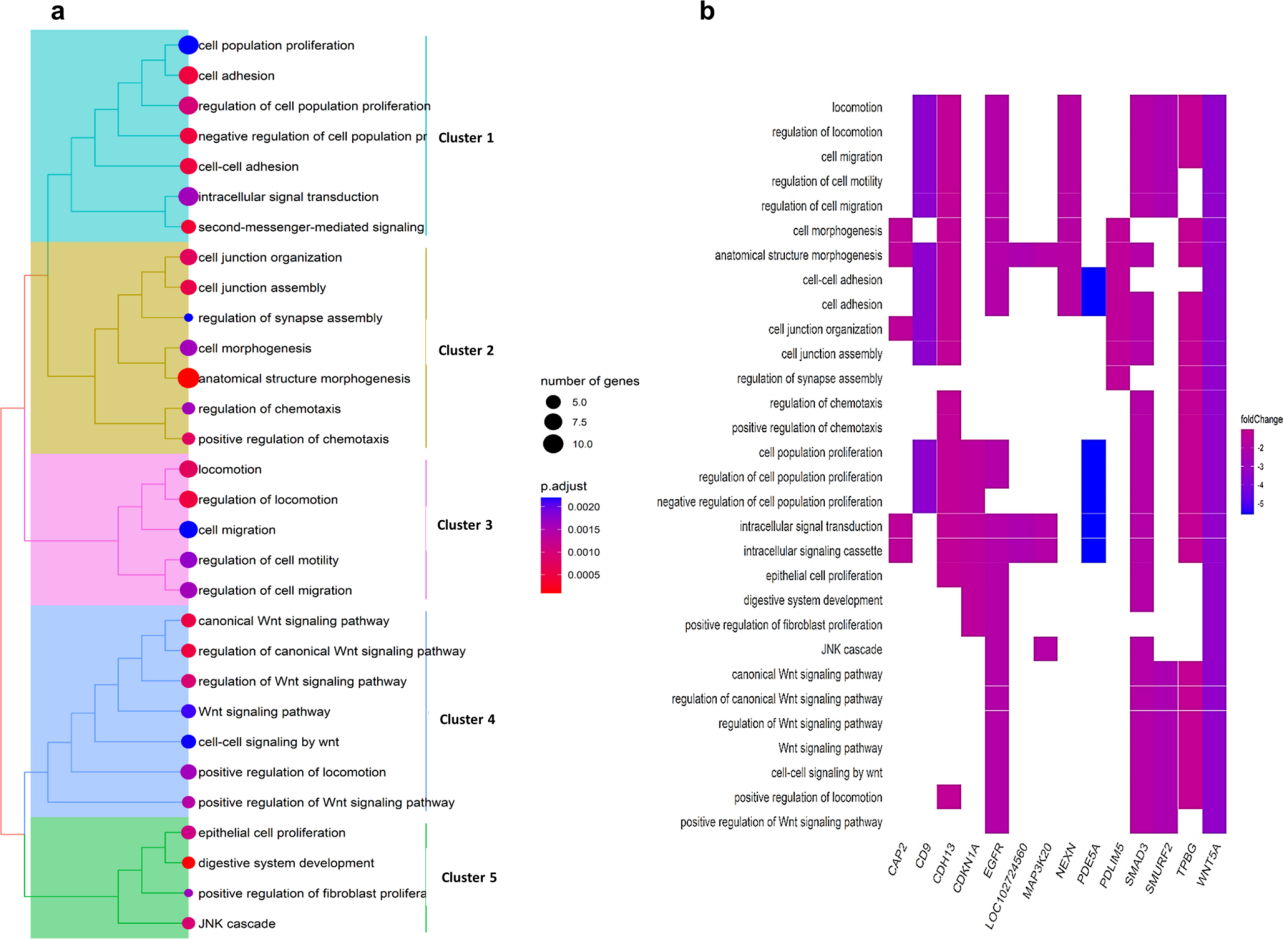
GO enrichment analysis of downregulated biological processes from the comparison of HDFa versus DPSCs. Pathways with integrated significant proteins were visualized. **a** Tree plot with the color gradient related to the *P*-value level of significance. **b** Heat plot with the color gradient related to the log_2_ fold change. Clusters 1–5 represent grouped pathways: #1 proliferation, adhesion, and signal transduction; #2 cell junction, chemotaxis, and morphogenesis; #3 locomotion, migration, and motility; #4 Wnt signaling pathway; #5 proliferation, JNK cascade, and digestive system development

**Fig. 5:**
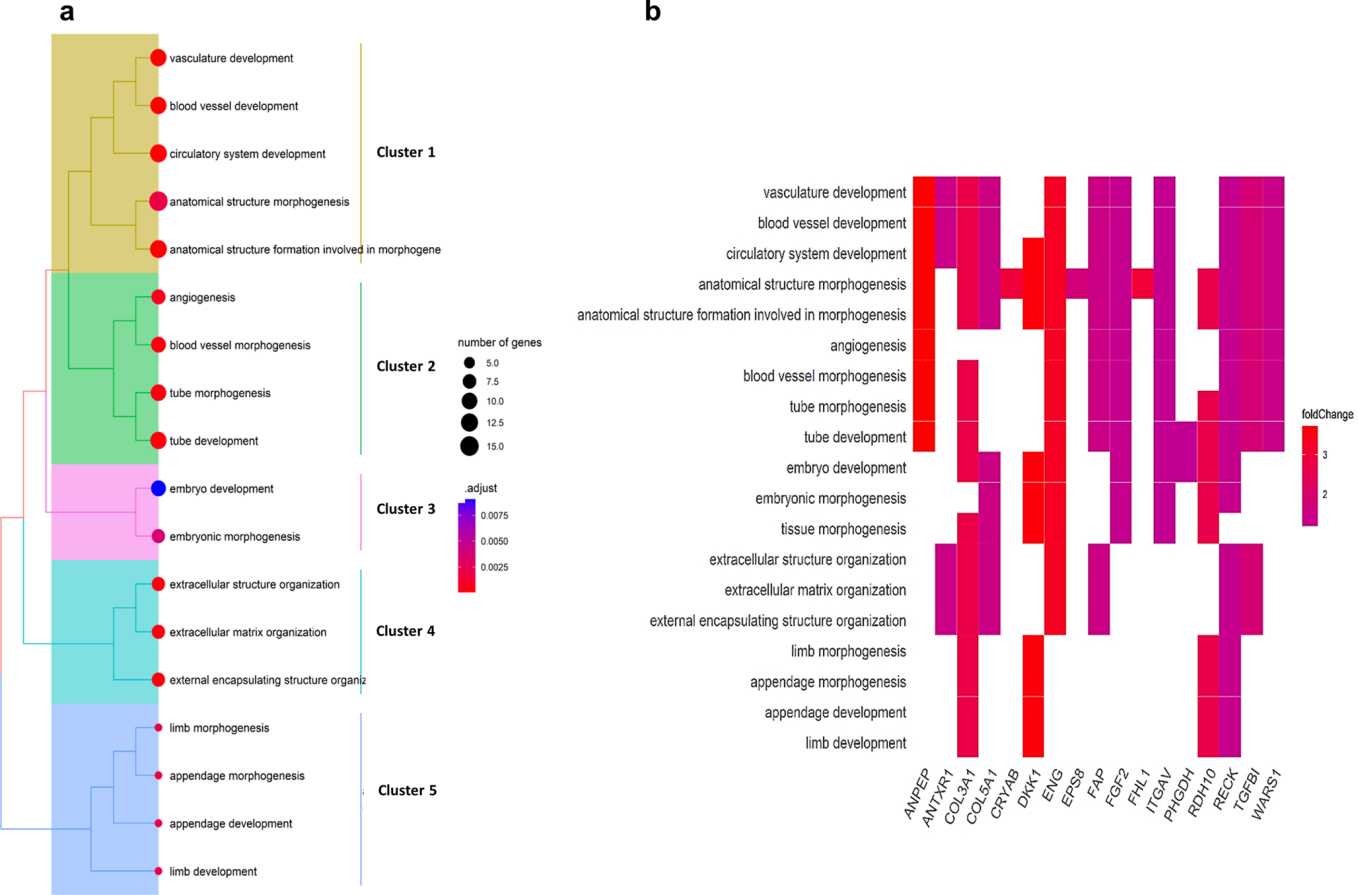
GO enrichment analysis of upregulated biological processes from the comparison of AD-MSCs versus DPSCs. Pathways with integrated significant proteins were visualized. **a** Tree plot with the color gradient related to the *P*-value level of significance. **b** Heat plot with the color gradient related to the log_2_ fold change. Clusters 1–5 represent grouped pathways: #1 circulatory system development, #2 angiogenesis, #3 embryonic development, #4 extracellular structure organization, and #5 appendage development

For other comparisons, statistically significant proteins were included in pathways, which are depicted in the form of a tree plot (Fig. S3a and Fig. S4a). Within the tree plot, individual pathways were clustered. Proteins with corresponding abundances that were included in the pathways are depicted in a heat map (Fig. S3b and Fig. S4b). Accumulated/depleted proteins in the heat maps are visualized in relation to HDFa versus DPSCs or AD-MSCs versus DPSCs. In the first case, GO enrichment revealed 47 biological processes (Fig. S3). Essential GO terms included canonical Wnt signaling pathway (GO:0060070, 3 accumulated and 5 depleted proteins), locomotion (GO:0040011, 11 accumulated and 8 depleted proteins), ossification (GO:0001503, 4 accumulated and 3 depleted proteins), tissue development (GO:0009888, 6 accumulated and 7 depleted proteins), regulation of BMP signaling pathway (GO:0030509, 2 accumulated and 2 depleted proteins), and regulation of MAP kinase activity (GO:0043405, 2 accumulated and 2 depleted proteins). For deeper insight into functional patterns we made GO enrichment separately for accumulated and depleted proteins, consequently only 30 biological processes downregulated in HDFa compared to DPSCs (Fig. 4). These processes were grouped into 5 clusters: #1 proliferation, adhesion, and signal transduction; #2 cell junction, chemotaxis, and morphogenesis; #3 locomotion, migration, and motility; #4 Wnt signaling pathway; #5 proliferation, JNK cascade, and digestive system development. In AD-MSCs versus DPSCs comparison, GO enrichment analysis revealed 18 biological processes (Fig. S4). Essential GO terms included extracellular matrix organization (GO:0030198, 7 accumulated, 2 depleted), regulation of canonical Wnt signaling pathway (GO:0060828, 3 accumulated, 4 depleted), vasculature development (GO:0001944, 11 accumulated, 1 depleted), and cell migration (GO:0016477, 11 accumulated, 8 depleted). Split GO enrichment analysis revealed only 18 biological processes upregulated in AD-MSCs compared to DPSCs (Fig. 5). These processes were grouped into 5 clusters: #1 circulatory system development, #2 angiogenesis, #3 embryonic development, #4 extracellular structure organization, and #5 appendage development.

### Functional Analysis of HDFa and Adult MSCs by GSEA

Next, we analyzed proteomic data using a complementary approach—gene set enrichment analysis (GSEA). This method allows detecting coordinated patterns of protein abundance changes. It analyzes ranked lists of proteins without pre-filtering based on statistical significance. By ranking proteins based on their log_2_ fold changes, GSEA evaluates whether specific protein sets are accumulated or depleted without a hard threshold. The authors suggested using the complete ranked gene list in GSEA because this approach captures subtle yet coordinated shifts in gene expression across a set of genes that may be overlooked when only statistically significant genes are analyzed, thereby better reflecting the underlying biological correlation with the phenotypic class distinction [34].

**Fig. 6:**
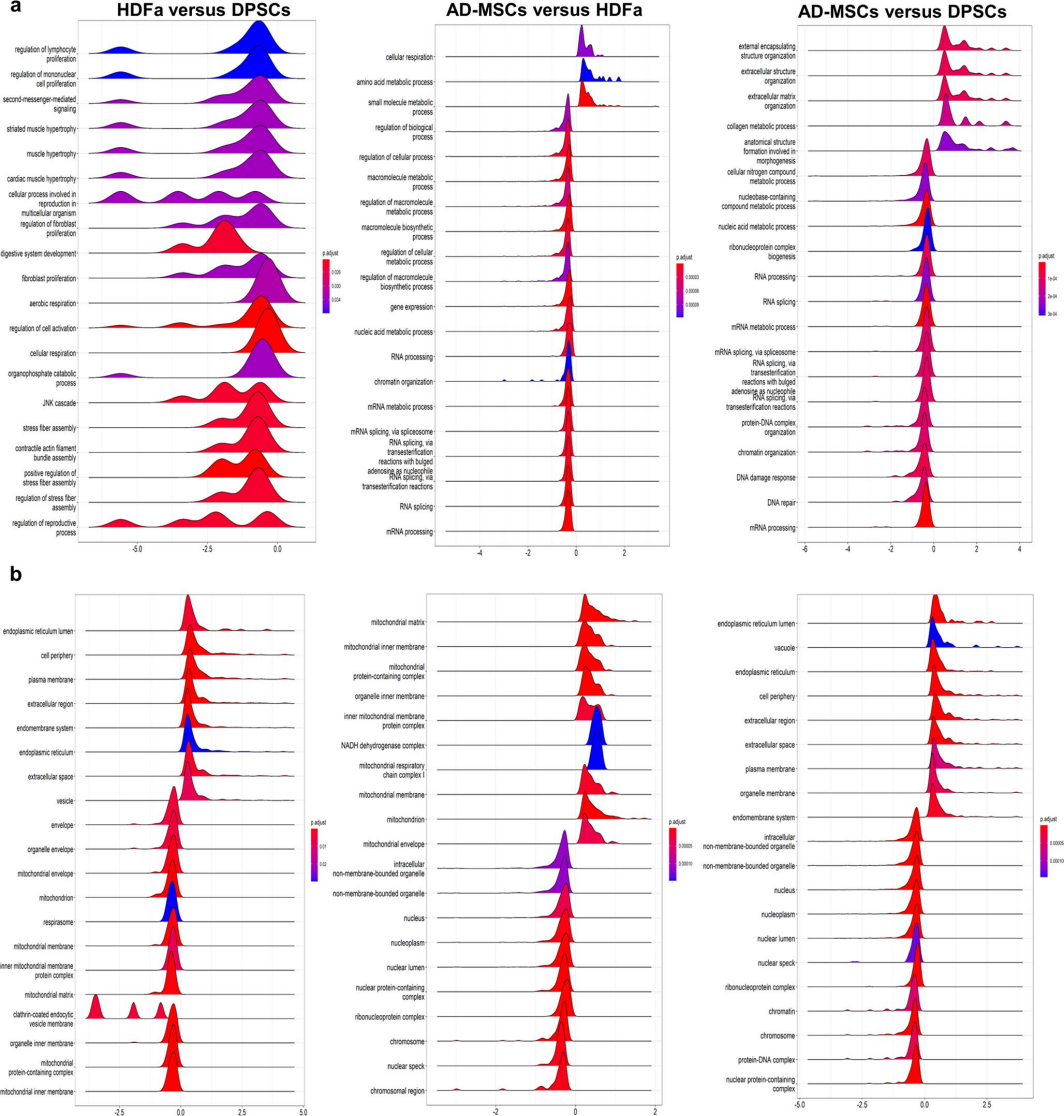
GSEA of divergently regulated protein groups in MSCs of different origins and HDFa **a** Biological processes. **b** Cellular localization. Results are presented as ridgeplots of the 20 most significant terms. The color gradient depicts the level of significance (adjusted *P*-value with the Benjamini–Hochberg method), and X-axis shows log_2_ fold change. The shape corresponds to the number of proteins with a specific fold change

Using GSEA, we determined that HDFa, compared to DPSCs, downregulated 20 dominant pathways, including proliferation, JNK cascade, digestive system development, and respiration (Fig. 6a). Such results partially correspond with GO enrichment reported in the previous subsection. From the localization perspective, accumulated proteins belonged to the plasma membrane, endoplasmic reticulum, and extracellular region/space. On the other hand, depleted proteins localized mainly in mitochondrial structures, such as envelope, respirasome, and inner membrane (Fig. 6b).

Comparison of HDFa versus AD-MSCs showed downregulated 3 pathways, namely cellular respiration, amino acid, and small molecule metabolic process. The location and shape of other pathways in ridgeplot are nearly identical, indicating comparable protein numbers with similar minimal fold change between cell types. Of note, proteins depleted in HDFa compared with AD-MSCs were involved mainly in several mitochondrion structures (Fig. 6). The common feature of HDFa compared to the two other cell types was the depletion of proteins involved in cellular respiration. Comparison of AD-MSCs versus DPSCs showed 5 upregulated pathways: external encapsulating structure organization, extracellular structure organization, extracellular matrix organization, collagen metabolic process, and anatomical structure formation involved in morphogenesis. The location and shape of other pathways in the ridgeplot were almost identical.

### HDFa Showed Consistent Abundance Ratios for 4 Proteins Compared with Other Cell Types

The next point of the study was to find a potential signature of HDFa—proteins with characteristic accumulation compared with other cell types. We revealed 4 such significantly different proteins: neurofilament medium polypeptide, tenascin, nicotinate-nucleotide pyrophosphorylase, and actin filament-associated protein 1. One of them, actin filament-associated protein 1, was less abundant in HDFa. Conversely, the remaining 3 accumulated in HDFa. The abundance ratios of these 4 proteins for HDFa versus alternative cell types were very similar (Table 1). We also determined signatures for AD-MSCs (9 proteins) and DPSCs (16 proteins).

**Table 1:**
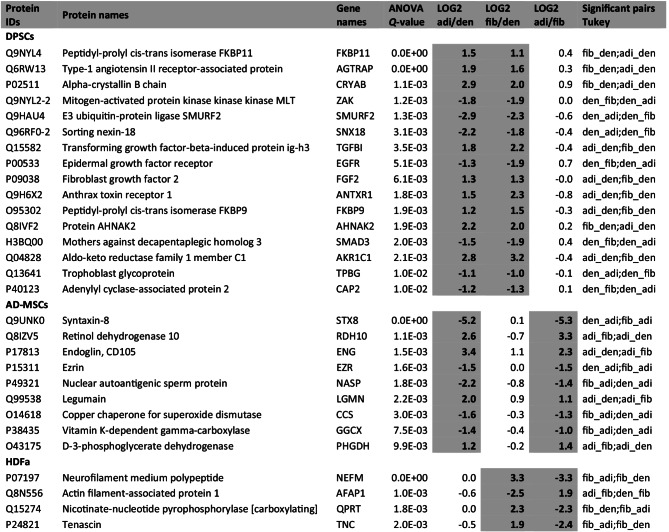
Description of signature proteins for HDFa or adult MSCs

Bold and shaded ratios indicate proteins with the same pattern for a specific type of cell; fib (HDFa), adi (AD-MSCs), and den (DPSCs)

### Verification of Three CD Markers Detected by LC-MS Analysis by Flow Cytometry and Western Blot

Flow cytometry is a method widely applicable in clinical practice [35], with the potential to distinguish cell origin [19]. For this reason, we screened for surface CD markers among differentially abundant proteins. We detected 3 CD markers, CD9 (tetraspanin), CD13 (aminopeptidase N), and CD105 (endoglin) by label-free LC-MS analysis.

**Fig. 7:**
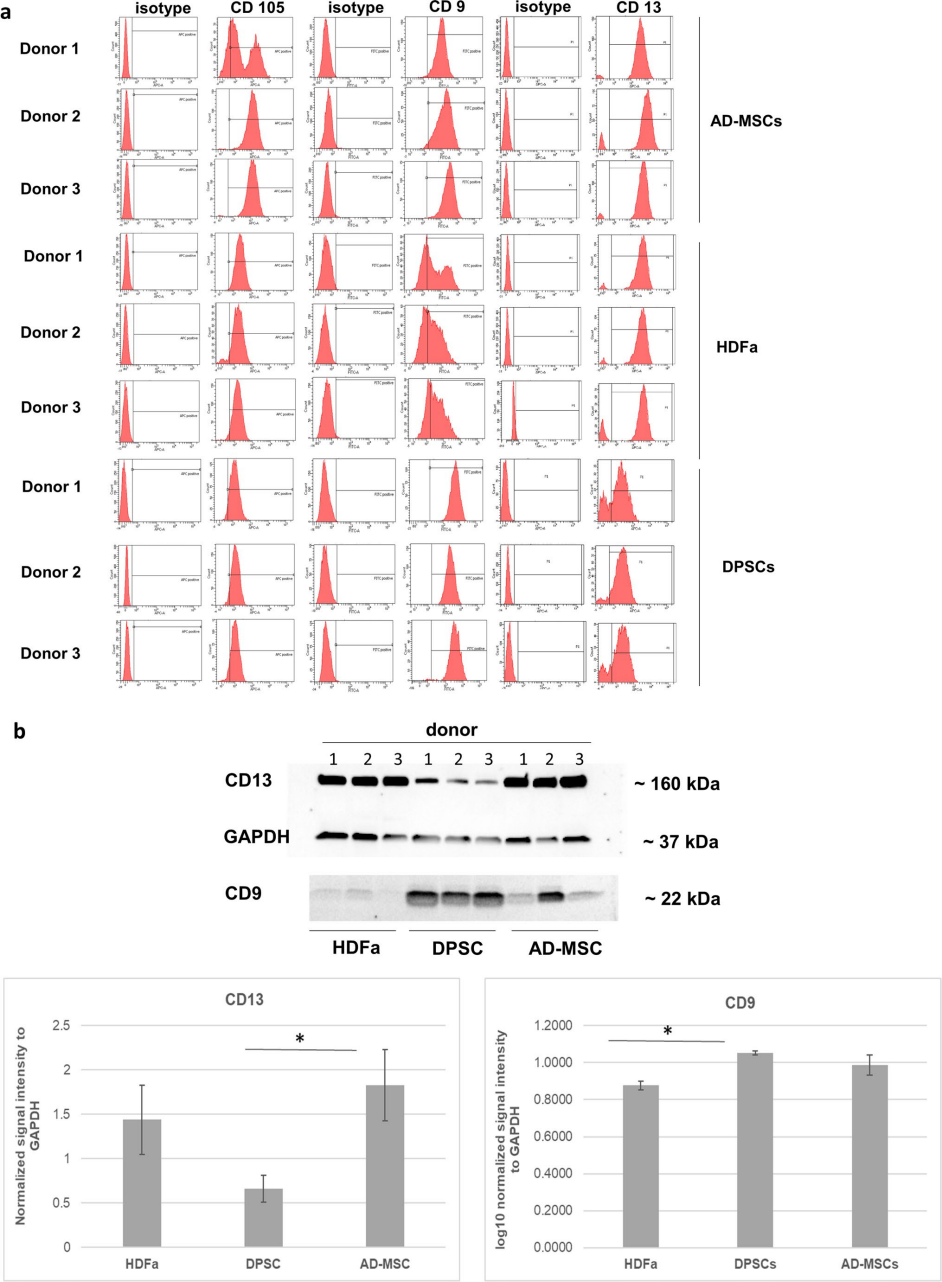
Verification of surface markers. **a** Flow cytometry histograms. **b** Western blot analysis of proteins (CD markers) stratifying HDFa and/or two types of MSCs. Normalized data are presented in the form of blots and column graphs as means ± SD, *n* = 3 per tissue source; * indicates statistical significance at *P* ≤ 0.05

The detection of the CD105 as a signature marker in proteomic analysis was somewhat puzzling, such as it showed a similar amount by flow cytometry in cells from different sources: 95.4% ± 3.3 of HDFa, 92.4% ± 2.9 of DPSCs, and 91.3% ± 11.6 of AD-MSCs. However, a closer evaluation of the flow cytometry histograms allowed to spot a signal shift to the right on the x-axis for AD-MSCs compared to the other 2 stem cell sources (Fig. 7a). In contrast, the CD9 marker showed an apparently shifted signal to the left on the x-axis of the flow cytometry histogram and a corresponding decreased marker presence in only 63.3% ± 2.1 of HDFa versus DPSCs (98.5% ± 1.6) and AD-MSCs (96.7% ± 1.1) but without statistical significance. Finally, the CD13 marker was specific for DPSCs with decreased signal (83.2% ± 8.2) in comparison to HDFa (92.4% ± 5.5) and AD-MSCs (93.9% ± 2.7) shifted to the left on the x-axis of the flow cytometry histogram again without statistical significance.

We further verified our results by detecting the tetraspanin and aminopeptidase N proteins using western blot analysis (Fig. 7b and c). Similarly to proteomic analysis, western blot confirmed a reduced signal intensity for CD13 (aminopeptidase N) in DPSCs compared to AD-MSCs and HDFa, with statistical significance (*P* = 0.050). CD9 (tetraspanin) for AD-MSCs showed high variability but could clearly distinguish HDFa from DPSCs in the proteomic dataset and by western blotting.

## Discussion

The ability of the human body to regenerate tissue decreases significantly with age. With the aging population, the pressure to find suitable therapies to treat defects is increasing. One form of effective therapy is regenerative medicine using MSCs. Over the past decades, MSCs have been frequently studied due to their undeniable clinical potential and minimal ethical constraints [36]. The isolation of MSCs from different tissues, their re-implantation, and the complex process of endogenous tissue repair in vivo are not yet fully understood. Alternative sources of MSCs are desirable with non-invasive collection as painless as possible for the patient. Of note, MSCs derived from different specific sources might show preferences in their differentiation potential. Besides, a vital question is: Which of the expressed genes/proteins/metabolites define the molecular signature of MSCs from a specific source and thus predict their therapeutic efficacy? At the same time, these molecules could serve as markers of a particular source of MSCs since the basic definition of MSCs according to ISCT is currently inadequate [37]. Comparative global studies of MSCs from different sources aspire to highlight differences between them and HDFa, including stratifying expressed genes, methylation status, accumulated proteins, and regulated signaling pathways. The authors [38] proposed the cellular identity for HDFa by defining their HOX code. Also, other studies identified several genes and metabolites as candidate HDFa markers [39, 40]. Integrative transcriptomic and proteomic analysis of BM-MSCs, AD-MSCs, and umbilical cord (UC)-MSCs showed source-specific regenerative signature—adult-MSCs facilitated extracellular matrix (ECM) remodeling and promoted angiogenesis [41].

Our study demonstrated how the tissue origin influenced the proteomic profile of MSCs and HDFa and suggested molecules, which can distinguish cell types. HDFa are not only a promising potential alternative to MSCs, but they often contaminate tissue isolates and thus potentially complicate clinical application, for example, by decreasing cellular differentiation [42]. Therefore, the characterization of HDFa has practical significance. We revealed 86 differentially abundant proteins across 3 cell types. Next, we used two complementary approaches to analyze affected signaling pathways: GO enrichment and GSEA. GO enrichment comparing HDFa versus DPSCs revealed that pathways in HDFa were predominantly downregulated. These pathways were grouped into 5 clusters. One cluster includes pathways that are associated with cell adhesion, a process that is connected to many aspects of tissue physiology, including cell differentiation, migration, and survival [43]. Another essential cluster is the canonical Wnt signaling pathway, an important factor in stem cell biology. It affects the self-renewal of stem cells in various tissues. Also, this pathway regulates proliferation, differentiation, and maturation. In addition, Wnt signaling is crucial for bone formation and thus represents a potential target for the development of therapeutics [44, 45]. Cluster 5, included JNK signaling, epithelial cell proliferation, and fibroblast proliferation pathways. In fact, MSCs are known for their strong immunosuppressive and differentiation properties. Systemic and local MSC therapy was used to treat intestinal diseases in more than 1500 patients in phase I and II studies. Particularly, bone marrow and AD-MSCs were used [46, 47]. Similarly, the substantial role of intestinal fibroblasts both in the maintenance of epithelial and immune homeostasis in the intestine and the response to tissue damage was confirmed [48]. In conclusion, HDFa, compared to DPSCs, downregulated pathways responsible for cell migration, adhesion, and proliferation, necessary during tissue repair. Thus, they might be less suitable for tissue regeneration.

GO enrichment analysis comparing AD-MSCs versus DPSCs determined that pathways in AD-MSCs were predominantly upregulated. Most of the identified signaling pathways contribute to angiogenesis, vascularization, and extracellular matrix remodeling. Also, GSEA showed upregulated pathways involved in extracellular structure organization and collagen remodeling. These results corroborate previous studies that define AD-MSCs as particularly suitable for promoting angiogenesis and neovascularization. Functional vascular networks are vital for future clinical applications. In fact, they depend on the neovascularization potential of the graft [49].

The proteomic analysis also determined three cell surface markers differentially abundant in cells from various tissue origins. CD105 is primarily involved in blood vessel development [50]. Our data indicated that AD-MSCs could be superior for angiogenesis and vascularization. MSCs from both sources and also from HDFa revealed more than 90% presence of CD105 (apart from one donor-derived AD-MSCs) but with a different shift on X-axis of the histogram. This finding is useful for the discrimination of AD-MSCs. Another group reported similar results: The authors proposed discriminatory cell surface markers for foreskin fibroblasts and MSCs of different origins [19]. They determined a decreased percentage of positivity of CD105 for foreskin fibroblasts compared to other MSCs.

CD9, tetraspanin is a cell-surface protein and a member of the tetraspanin family, which is present in several tissues. It modifies multiple cellular events, including adhesion, migration, proliferation, and survival. Its reduced abundance in HDFa fully corresponded with enrichment analyses, where HDFa, compared to DPSCs, showed downregulated pathways associated with migration, adhesion, and proliferation.

CD13, aminopeptidase N belongs to the group of moonlighting enzymes. It is expressed in various cells/tissues and is associated with proliferation, differentiation, migration, angiogenesis, invasion, vasoconstriction, and the regulation of normal and impaired immune function [51]. The authors showed that CD13 regulates FAK activation to promote MSC adhesion, migration, and tissue repair. Consequently, they suggested that CD13 could be a viable target to enhance the efficacy of MSC therapies [52]. We detected CD13 depleted in DPSCs compared to AD-MSCs and HDFa. Enrichment analysis pointed that DPSCs could be less suitable for angiogenic and vascularization therapy than AD-MSCs.

However, this research is not without limitations. Our study was conducted on 3 biological samples from each cell origin. A follow-up investigation on a larger cohort of donors is preferable to better account for plausible individual variability in cellular characteristics and to confirm stratifying protein markers. Our study was performed on passage 5. Many studies used passages 3‒5 [53–55]. Moreover, MSCs have limited proliferative potential and can undergo senescence after several passages in vitro, diminishing their regenerative capacity [56]. On the other hand, for regenerative therapy, it is necessary to prepare a lot of cells. These limitations necessitate further research to optimize MSC culture and multiplication techniques, as well as to identify the most suitable sources of MSCs for specific therapeutic applications [57, 58]. Also, a growing number of studies suggest that MSCs are highly heterogeneous populations containing cells with different multipotency properties, progenitors, and states [2, 57]. For example, we observed that AD-MSCs from one donor showed an atypical presence of flow cytometry marker CD105. Furthermore, additional studies are needed to ensure that the results observed in vitro can be translated into medical applications.

## Conclusion

Proteomics allowed to identify the proteins and mechanisms associated with different sources of MSCs and HDFa. Our findings indicate that proteomics can enhance understanding of the complex characteristics of MSCs. Our results point to HDFa as a suitable alternative to MSCs, with specific distinguishing characteristics. They showed a minimum of differentially accumulated proteins compared to AD-MSCs. Compared to DPSCs, they might be a less suitable source for treating defects, as HDFa displayed downregulated signaling pathways responsible for adhesion, proliferation, or migration. In contrast, AD-MSCs confirmed their properties as the most suitable source for angiogenesis. Tissue repair requires a well-organized integration of complex biological and molecular events: cell migration and proliferation, extracellular matrix deposition, angiogenesis, and remodeling. Selecting MSCs from suitable tissue origin, cultivation, and expansion conditions can support tissue regeneration.

## Electronic Supplementary Material

Below is the link to the electronic supplementary material.

Supplementary Material 1

Supplementary Material 2

## Data Availability

All data analyzed during this study are included in this published article and its supplementary materials. The mass spectrometry raw data and proteomic processing are available in the ProteomeXchange Consortium via the PRIDE partner repository [59] with the dataset identifier PXD061092 and DOI: 10.6019/PXD061092.
