# Supplementary material for: Proteomic Study Revealed a Distinction Between Human Dermal Fibroblasts and Mesenchymal Stem Cells from Different Sources
Source: Stem Cell Rev Rep. 2025 Jun 27;21(7):2237–52. doi: 10.1007/s12015-025-10926-4 (PMC12408728; doi:10.1007/s12015-025-10926-4)
Supplement: Supplementary file 1 — Supplementary Material 1 [file 12015_2025_10926_MOESM1_ESM.pptx]

## Slide 1
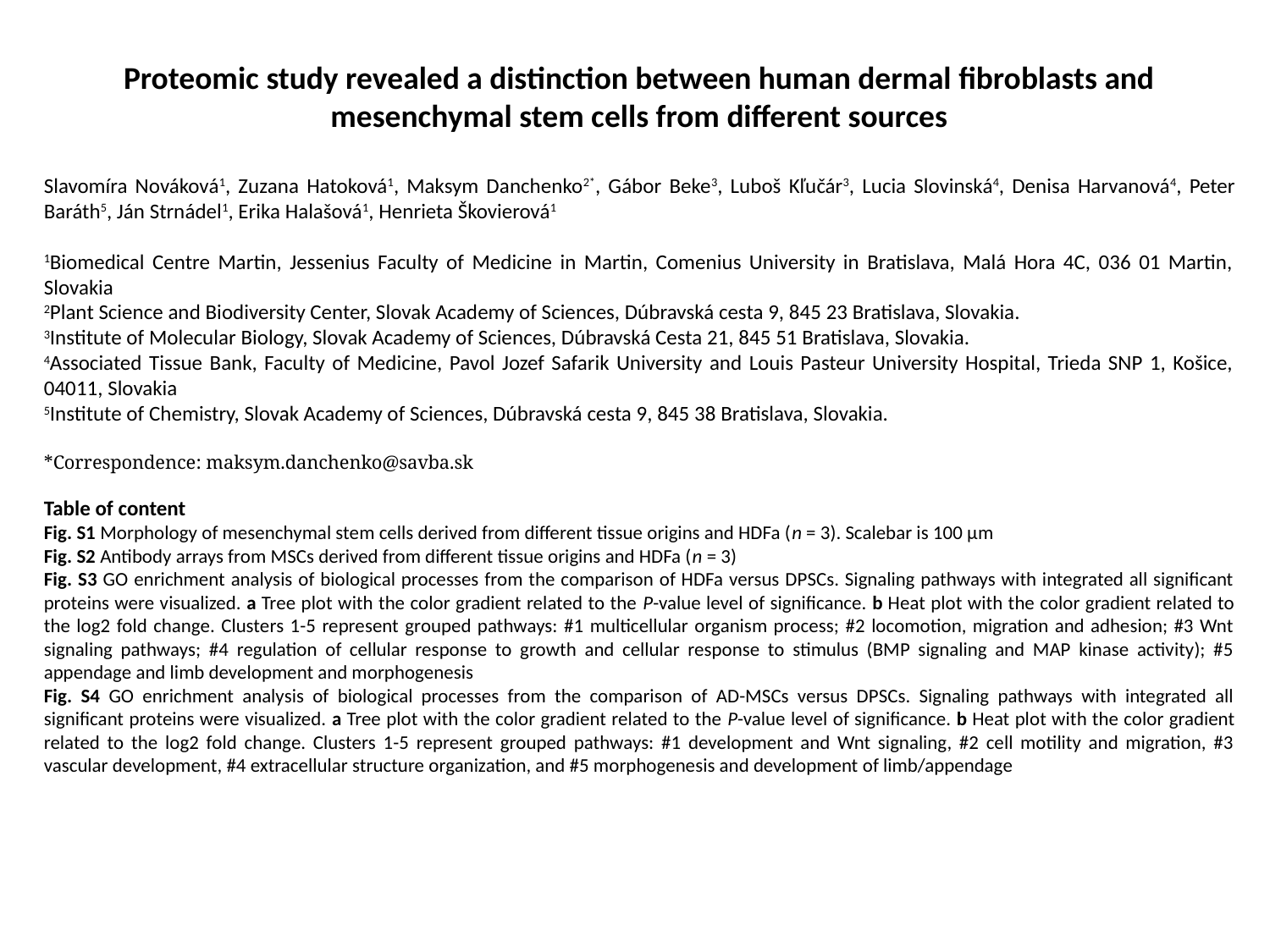

Proteomic study revealed a distinction between human dermal fibroblasts and mesenchymal stem cells from different sources
Slavomíra Nováková1, Zuzana Hatoková1, Maksym Danchenko2*, Gábor Beke3, Luboš Kľučár3, Lucia Slovinská4, Denisa Harvanová4, Peter Baráth5, Ján Strnádel1, Erika Halašová1, Henrieta Škovierová1
1Biomedical Centre Martin, Jessenius Faculty of Medicine in Martin, Comenius University in Bratislava, Malá Hora 4C, 036 01 Martin, Slovakia
2Plant Science and Biodiversity Center, Slovak Academy of Sciences, Dúbravská cesta 9, 845 23 Bratislava, Slovakia.
3Institute of Molecular Biology, Slovak Academy of Sciences, Dúbravská Cesta 21, 845 51 Bratislava, Slovakia.
4Associated Tissue Bank, Faculty of Medicine, Pavol Jozef Safarik University and Louis Pasteur University Hospital, Trieda SNP 1, Košice, 04011, Slovakia
5Institute of Chemistry, Slovak Academy of Sciences, Dúbravská cesta 9, 845 38 Bratislava, Slovakia.
*Correspondence: maksym.danchenko@savba.sk
Table of content
Fig. S1 Morphology of mesenchymal stem cells derived from different tissue origins and HDFa (n = 3). Scalebar is 100 µm
Fig. S2 Antibody arrays from MSCs derived from different tissue origins and HDFa (n = 3)
Fig. S3 GO enrichment analysis of biological processes from the comparison of HDFa versus DPSCs. Signaling pathways with integrated all significant proteins were visualized. a Tree plot with the color gradient related to the P-value level of significance. b Heat plot with the color gradient related to the log2 fold change. Clusters 1-5 represent grouped pathways: #1 multicellular organism process; #2 locomotion, migration and adhesion; #3 Wnt signaling pathways; #4 regulation of cellular response to growth and cellular response to stimulus (BMP signaling and MAP kinase activity); #5 appendage and limb development and morphogenesis
Fig. S4 GO enrichment analysis of biological processes from the comparison of AD-MSCs versus DPSCs. Signaling pathways with integrated all significant proteins were visualized. a Tree plot with the color gradient related to the P-value level of significance. b Heat plot with the color gradient related to the log2 fold change. Clusters 1-5 represent grouped pathways: #1 development and Wnt signaling, #2 cell motility and migration, #3 vascular development, #4 extracellular structure organization, and #5 morphogenesis and development of limb/appendage

## Slide 2
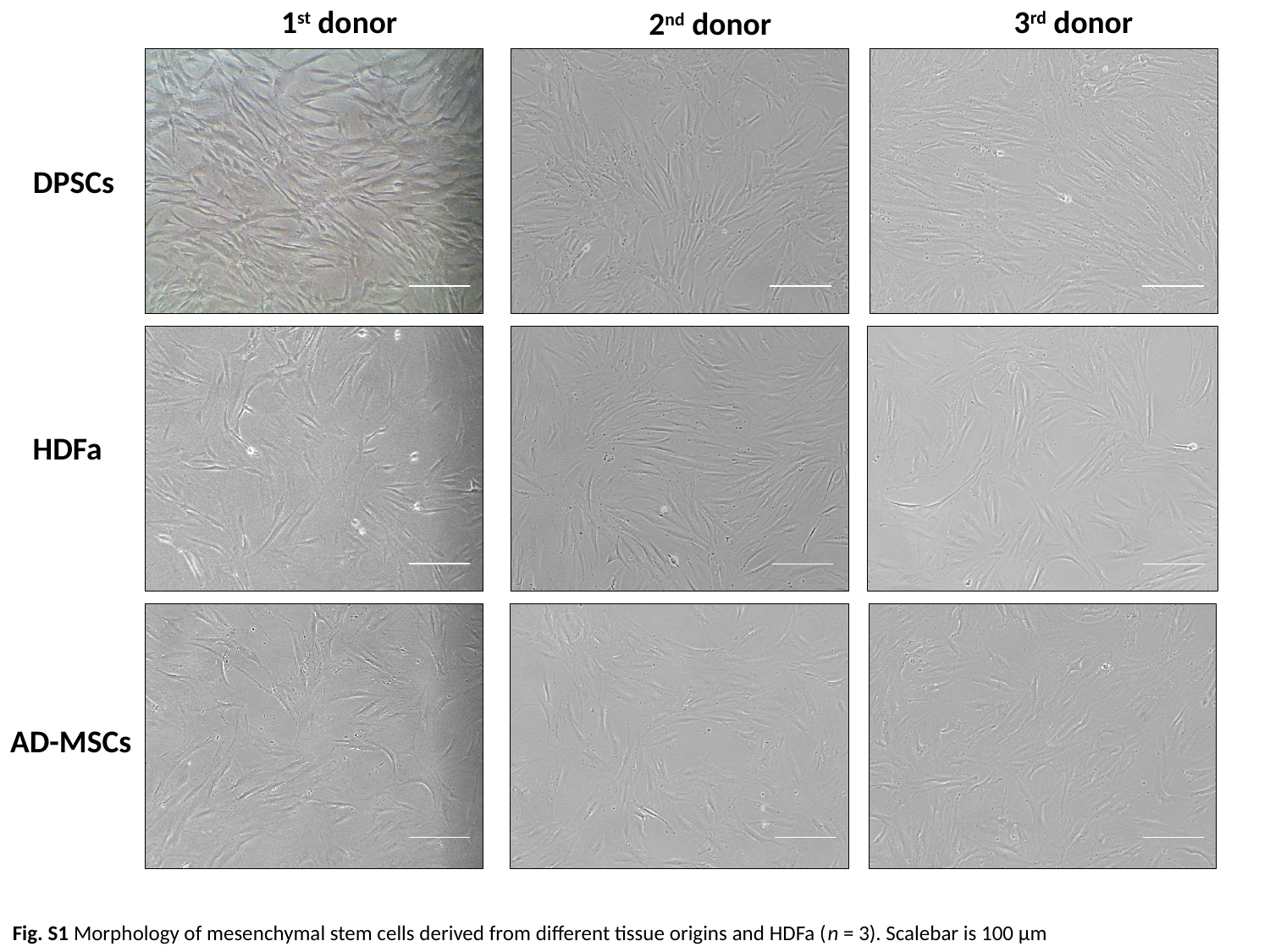

1st donor
3rd donor
2nd donor
DPSCs
HDFa
AD-MSCs
Fig. S1 Morphology of mesenchymal stem cells derived from different tissue origins and HDFa (n = 3). Scalebar is 100 µm

## Slide 3
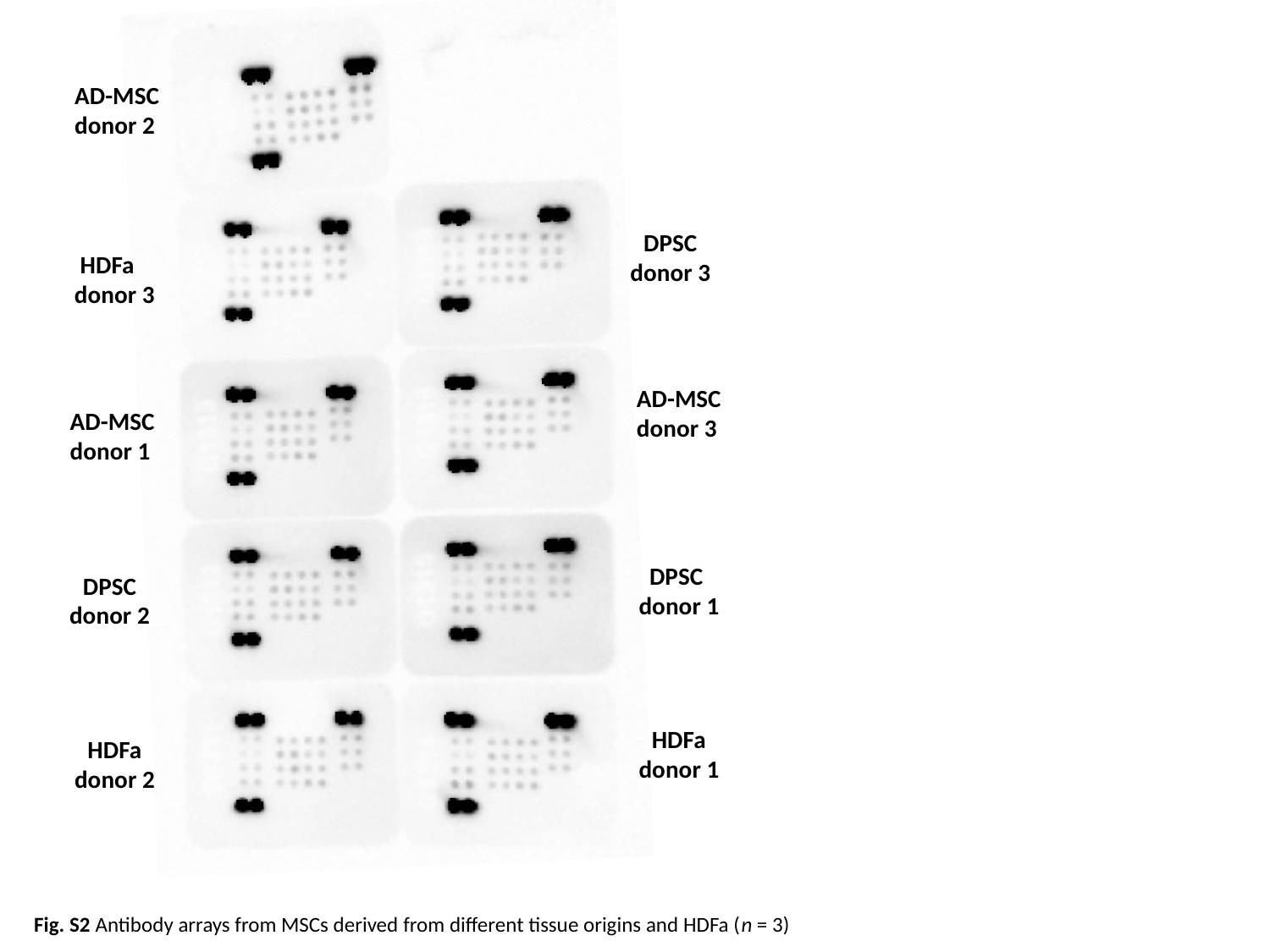

AD-MSC
donor 2
DPSC
donor 3
 HDFa
donor 3
AD-MSC
donor 3
AD-MSC
donor 1
DPSC
 donor 1
DPSC
donor 2
HDFa
donor 1
HDFa
donor 2
Fig. S2 Antibody arrays from MSCs derived from different tissue origins and HDFa (n = 3)

## Slide 4
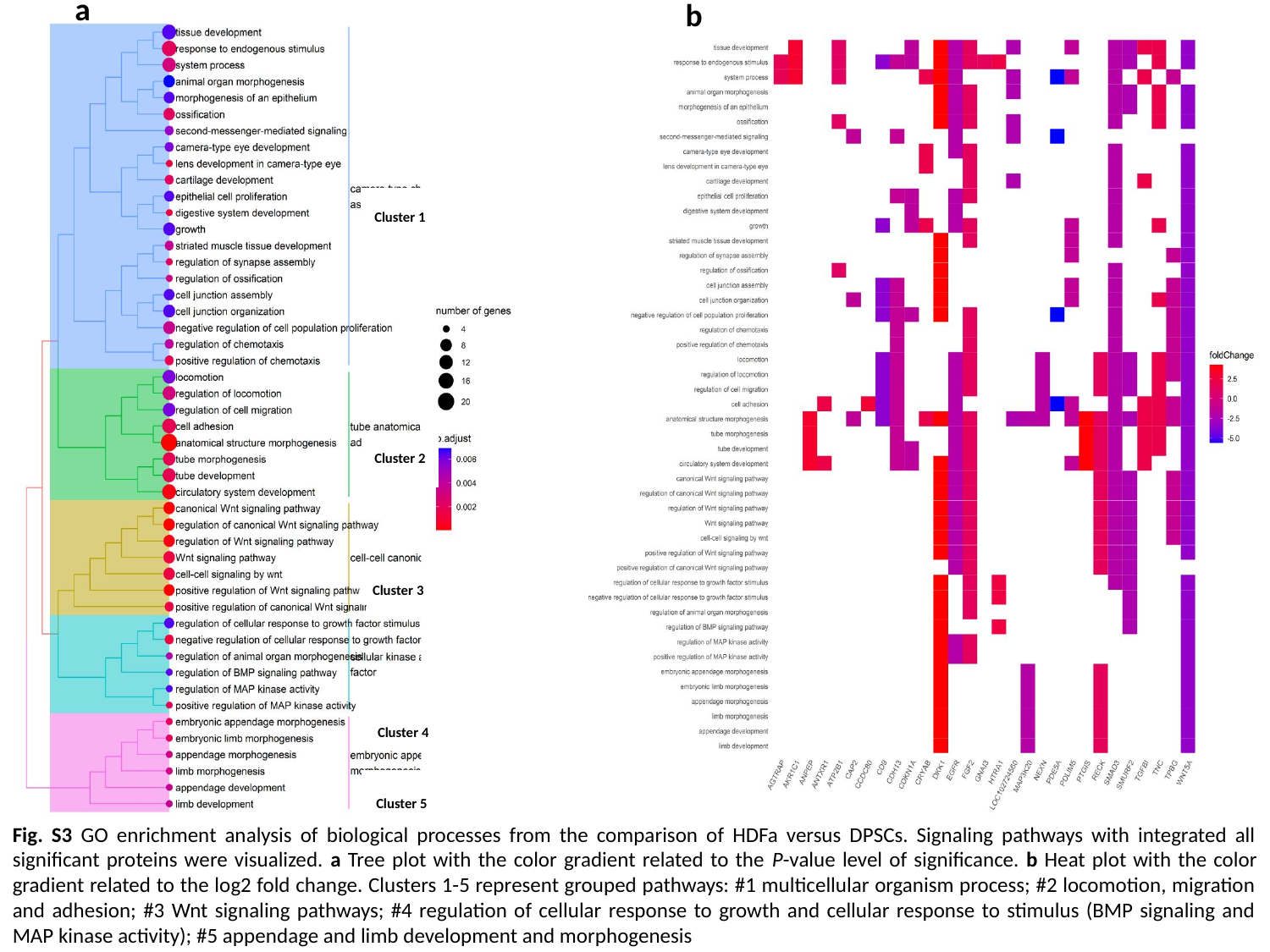

a
b
Cluster 1
Cluster 2
Cluster 3
Cluster 4
Cluster 5
Fig. S3 GO enrichment analysis of biological processes from the comparison of HDFa versus DPSCs. Signaling pathways with integrated all significant proteins were visualized. a Tree plot with the color gradient related to the P-value level of significance. b Heat plot with the color gradient related to the log2 fold change. Clusters 1-5 represent grouped pathways: #1 multicellular organism process; #2 locomotion, migration and adhesion; #3 Wnt signaling pathways; #4 regulation of cellular response to growth and cellular response to stimulus (BMP signaling and MAP kinase activity); #5 appendage and limb development and morphogenesis

## Slide 5
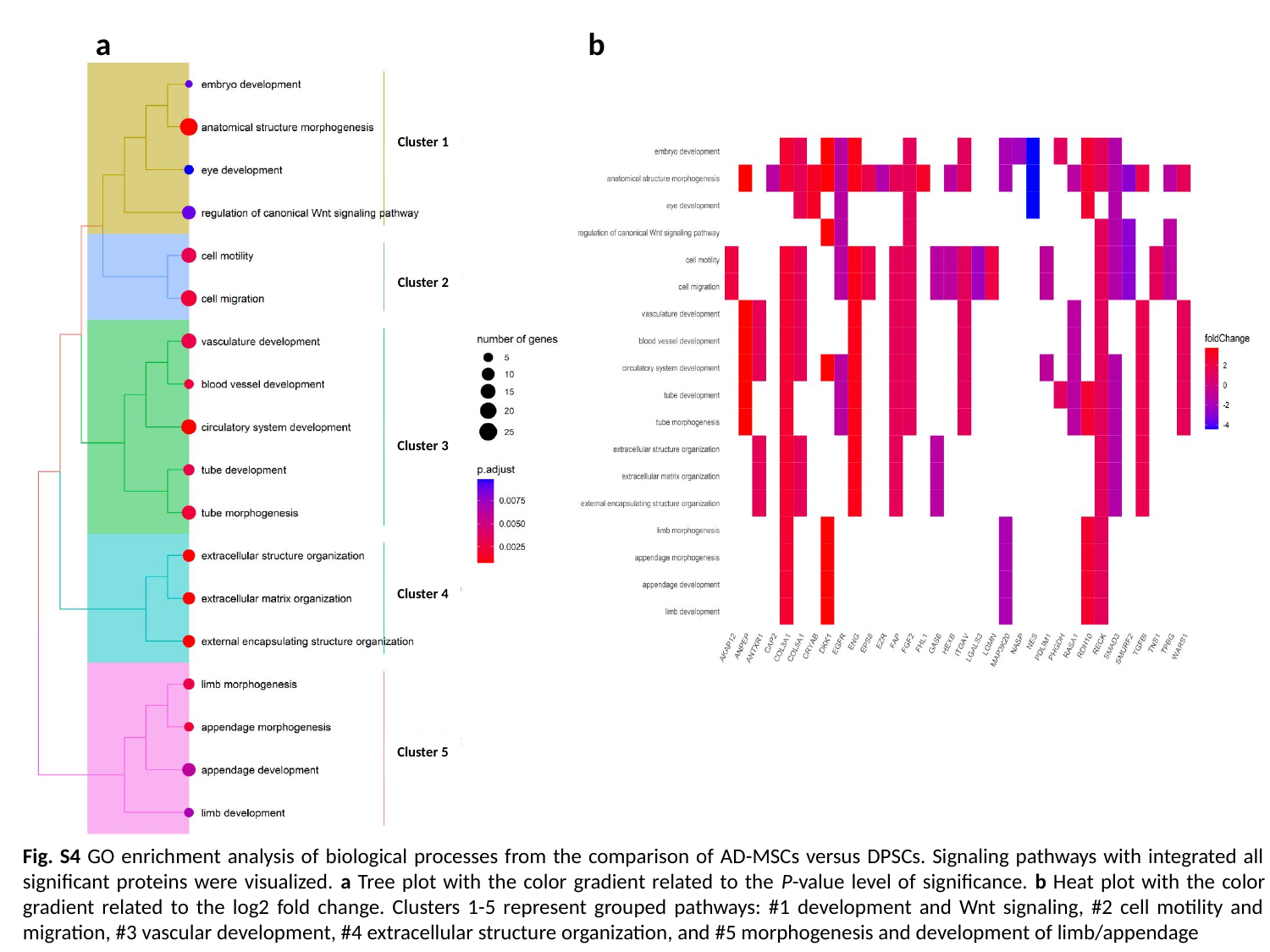

a
b
Cluster 1
Cluster 2
Cluster 3
Cluster 4
Cluster 5
Fig. S4 GO enrichment analysis of biological processes from the comparison of AD-MSCs versus DPSCs. Signaling pathways with integrated all significant proteins were visualized. a Tree plot with the color gradient related to the P-value level of significance. b Heat plot with the color gradient related to the log2 fold change. Clusters 1-5 represent grouped pathways: #1 development and Wnt signaling, #2 cell motility and migration, #3 vascular development, #4 extracellular structure organization, and #5 morphogenesis and development of limb/appendage
